# High-resolution 3D imaging uncovers organ-specific vascular control of tissue aging

**DOI:** 10.1126/sciadv.abd7819

**Published:** 2021-02-03

**Authors:** Junyu Chen, Unnikrishnan Sivan, Sin Lih Tan, Luciana Lippo, Jessica De Angelis, Rossella Labella, Amit Singh, Alexandros Chatzis, Stanley Cheuk, Mino Medhghalchi, Jesus Gil, Georg Hollander, Brian D. Marsden, Richard Williams, Saravana K. Ramasamy, Anjali P. Kusumbe

**Affiliations:** 1 Tissue and Tumor Microenvironments Group, Kennedy Institute of Rheumatology, University of Oxford, Oxford OX3 7FY, UK.; 2 Department of Prosthodontics, State Key Laboratory of Oral Diseases, West China Hospital of Stomatology, Sichuan University, Chengdu 610041, China.; 3 Heidelberg University Biochemistry Center, Im Neuenheimer Feld 328, Heidelberg D-69120, Germany.; 4 Weatherall Institute of Molecular Medicine, University of Oxford, Oxford, UK.; 5 Department of Rheumatology and Inflammation Research, University of Gothenburg, Gothenburg, Sweden.; 6 Kennedy Institute of Rheumatology, University of Oxford, Oxford OX3 7FY, UK.; 7 Institute of Clinical Sciences, Imperial College London, London W12 0NN, UK.; 8 MRC London Institute of Medical Sciences, Imperial College London, London W12 0NN, UK.; 9 Structural Genomics Consortium, NDM, University of Oxford, Oxford OX3 7DQ, UK.

## Abstract

Blood vessels provide supportive microenvironments for maintaining tissue functions. Age-associated vascular changes and their relation to tissue aging and pathology are poorly understood. Here, we perform 3D imaging of young and aging vascular beds. Multiple organs in mice and humans demonstrate an age-dependent decline in vessel density and pericyte numbers, while highly remodeling tissues such as skin preserve the vasculature. Vascular attrition precedes the appearance of cellular hallmarks of aging such as senescence. Endothelial VEGFR2 loss-of-function mice demonstrate that vascular perturbations are sufficient to stimulate cellular changes coupled with aging. Age-associated tissue-specific molecular changes in the endothelium drive vascular loss and dictate pericyte to fibroblast differentiation. Lineage tracing of perivascular cells with inducible PDGFRβ and NG2 Cre mouse lines demonstrated that increased pericyte to fibroblast differentiation distinguishes injury-induced organ fibrosis and zymosan-induced arthritis. To spur further discoveries, we provide a freely available resource with 3D vascular and tissue maps.

## Teaser

3D tissue imaging reveals maladaptations of blood vessels in organ aging, fibrosis, and arthritis.

## INTRODUCTION

Aging is linked to gradual degeneration of cellular and physiological integrity and loss of tissue and organ function. This deterioration is the leading risk factor for major diseases such as cancer and cardiovascular disease (*1*). Mammalian aging has been intensively investigated at the cellular level, leading to the identification of cellular hallmarks contributing to the aging process. These hallmarks include genomic instability, telomere shortening, epigenetic alterations, loss of proteostasis, deregulated nutrient sensing, mitochondrial dysfunction, cellular senescence, and stem cell exhaustion (*2*–*4*). Although such typical cell-intrinsic factors and their correlation to the aging process are well defined, the microenvironmental triggers and age-dependent perturbations at the tissue level remain poorly understood.

Across different organs, blood vessels are an essential component in maintaining tissue function and form transport routes for the circulation of cargo such as cells, oxygen, nutrients, or waste products (*5*, *6*). Blood vessels also provide inductive factors, termed angiocrine signals. These are essential for the survival and maintenance of stem/progenitor cells and other cell types within tissues (*7*, *8*).

Aging alters the cellular composition and architecture of tissues, thereby negatively affecting upon tissue function. However, vascular alterations occurring with aging across several organs remain unknown. In the skeletal system, age-dependent perturbations of the vascular niches trigger the loss of functional hematopoietic stem cells and osteoprogenitors (*9*, *10*). Moreover, recent pieces of evidence support the notion that extrinsic signals from the microenvironment are essential drivers of stem cell and tissue aging (*11*, *12*). Thus, elucidating the age-dependent tissue alterations, particularly of the blood vessels and their vascular niches, holds the potential to identify and target microenvironmental triggers regulating the aging process.

Here, we investigated organ-specific and age-dependent perturbations across different organs and tissues. Our analysis reveals that pericyte to fibroblast differentiation is a primary hallmark of tissue and organ aging.

## RESULTS

### Heterogeneity of vascular microenvironments across organs and age

To understand the age-related changes in tissue microenvironments, we tested ~100 antibodies for mapping endothelial cell (EC), pericyte, stromal cell, and matrix to understand their tissue-wide distribution in selected specimens. We then selected ~50 of these antibodies that were most informative in defining vascular microenvironments and extended the analysis across the kidney, rectus femoris muscle, spleen, thymus, liver, lung, uterus, heart, bladder, brain, skin, and gut. Thick longitudinal sections spanning these organs and tissues were generated and subjected to multiplex immunolabeling by the pairing of primary antibodies (table S1) with optimal fluorophore-tagged secondary antibody combinations. We provide full information on the working conditions and cell types marked by these antibodies and exemplar images of the negative controls (table S1 and fig. S1A).

**Fig. 1:**
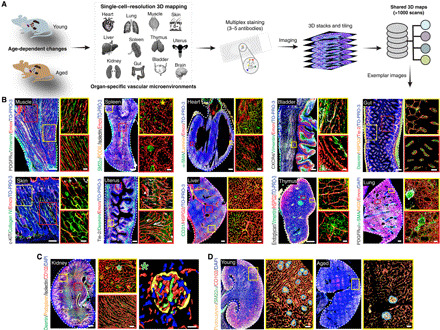
Single-cell–resolution 3D imaging of vascular microenvironments and the image database. (**A**) Schematic illustration of organs analyzed in young and aged wild-type (WT) mice. Multiplex staining (three to five antibodies) was performed, and whole-organ tile-scan images were acquired by confocal microscopy. More than ~1000 scans were acquired during the study with all the raw data available as a shared database. (**B**) Representative images of muscle, spleen, heart, bladder, gut, skin, uterus, liver, thymus, and lung from young mice stained with the antibodies as indicated. Insets on the right show high magnification of specific areas in the different organs. (**C**) Representative single-cell–resolution 3D images of a young mouse kidney stained with Desmin, Podoplanin, Isolectin, and CD102. Insets (middle) show high magnification of specific regions in the kidney. Asterisk on the right indicates higher magnifications with surface rendering for all markers. (**D**) Exemplar 3D images of young and aged kidney immunostained with Podocalyxin, SM22α, and CD102. Insets show higher magnifications with blood vessel surface rendered blue outlines around renal corpuscles (RC). Nuclei: TO-PRO-3 or DAPI as indicated. Scale bars, (B to D) 200 μm for tile scans of muscle, uterus, bladder, skin, gut, and thymus; 400 μm for spleen, kidney, heart, lung, and liver; insets, 50 μm; and single-cell–resolution images, 10 μm.

EC markers Endomucin (Emcn), Endoglin (CD105), CD31 (PECAM1), and CD102 (ICAM2) were used for vessel density analysis; also, other cell surface markers such as Tie-2, Bcam1, VEGFR2 (vascular endothelial growth factor receptor 2), and Podocalyxin were used to image and analyze blood vessels. α–Smooth muscle actin (α-SMA) staining was used for artery quantifications, and pericytes were identified by platelet-derived growth factor receptor β (PDGFRβ) and neural/glial antigen 2 (NG2). To image fibroblasts in young and aged tissues, fibroblast-specific protein-1 (FSP1) and Podoplanin antibodies were used. HSPG2, Laminin, Collagen IV, and Vimentin were used for identifying matrix. Desmin, Decorin, PDGFRα, and SM22α were used to image mesenchymal cells. Three-dimensional (3D) tile scans of sections were generated on a laser scanning confocal microscope with a single-cell resolution (Fig. 1, A and B). Exemplar tile scans from different organs showed the organization, expression patterns, and spatial distributions of ECs, pericytes, and mesenchymal stromal cells and enabled the generation of 3D maps of vascular microenvironments (Fig. 1B). Subsequently, computational surface rendering was performed on regions of interest (ROIs), which provides single-cell–level information from tile-scan 3D images (Fig. 1C and fig. S1B). Further, these high-resolution images allowed the analysis of localization and cell-cell and cell-matrix interactions at a single-cell resolution (Fig. 1C and fig. S1B). For example, the structural heterogeneity of capillaries in cortex versus medulla regions was highlighted by tile scans of the kidney (Fig. 1C). As evidenced by these images, Desmin-expressing stromal cells were localized mainly to the medulla region but not to the cortex of the kidney (Fig. 1C). In contrast, Podoplanin was confined to the glomeruli region (Fig. 1C). Similarly, Isolectin expression was restricted to the cortex region (Fig. 1C). Such high-resolution tile-scan imaging was applied across various organs from young and aging mice to investigate the age-dependent changes. Exemplar 3D tile-scan images from kidneys derived from young versus aged mice exhibited age-dependent changes (Fig. 1D). Organ- and age-dependent heterogeneity was revealed through the analysis of 3D whole-organ tissue maps. More than 1000 of such 3D tissue maps (around 6 terabytes of data) are available as a free resource for download (table S2) and further analysis via the OMERO web interface (http://homeros.kennedy.ox.ac.uk/pub/chen-et-al-2021-3dOrgans).

The specific 3D organization and spatial distribution of different vascular cell types, collectively with cell-cell and cell-matrix interactions in their natural milieu, influence and determine their functions within an organ (*13*, *14*). For example, the thymus provides a model for organ aging with marked age-dependent changes in cellular composition and tissue architecture that lead to the reduction of thymus size and loss of its function to support T cell development (*15*). Blood vessels maintain the tissue-specific regional oxygenation status and metabolic microenvironments in the thymus, which include hypoxic compartments (*16*). Further, tissue-derived signals in the thymus regulate the growth of its vascular network, which, in turn, establishes the conditions for T cell development (*17*, *18*). A comprehensive examination of 3D maps of thymus from young mice in our thymus image database highlighted differences in capillary organization and distribution across the cortex and medulla regions (fig. S1, C to E). Immunostaining with numerous EC markers showed a higher blood vessel density in the cortex compared to the medulla (fig. S1, C and D). Further, the arterial branches entered the cortex and connected to the cortical capillaries (fig. S1C). In line with capillary distributions and density, hypoxia-inducible factor 1α (HIF1α) immunostaining demonstrated that the cortex was oxygenated while the medulla was hypoxic (fig. S1C). Another notable feature was that the medulla but not the cortex was populated with mesenchymal stromal cells expressing SM22α and Desmin and fibroblasts identified by expression of FSP1 and Podoplanin (fig. S1, D and E). Moreover, the medulla but not the cortex was rich in multiple extracellular matrix proteins such as Collagen IV and Laminin and the adhesion complex–associated proteins Vimentin and Vinculin (fig. S1, D and E). Thus, our single-cell–resolution 3D tissue maps with an analysis of numerous markers provided comprehensive information on the heterogeneity of regional extracellular matrix and cell distributions. Several of these vascular maps are presented in table S3.

### Loss of vascular and pericyte abundance in aging tissues in mice and humans

**Fig. 2:**
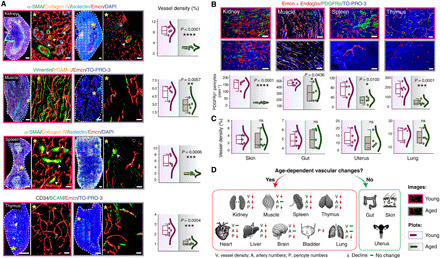
Loss of capillary, artery, and pericyte abundances in aging mice. (**A** and **B**) Representative 3D tile scans of young and aged kidney, muscle, spleen, and thymus with immunostaining as indicated. Arrowheads represent arteries. Asterisks denote higher-magnification insets showing surface-rendered capillaries. (A) The combo plots (right) showing vessel density quantifications (*n* = 5). (B) Combo plots (bottom) show quantifications of PDGFRβ^+^ pericytes in young and aged kidney, muscle, spleen (*n* = 5), and thymus (*n* = 6). (**C**) Combo plots show quantifications for vessel density in young and aged skin, gut, uterus, and lung (*n* = 5). (**D**) Diagram shows nine organs with age-dependent vascular decline and three organs without age-dependent vascular changes. The *P* value derived from two-tailed unpaired *t* tests is given for all graphs. ns, not significant, *P < 0.05; **P < 0.01; ***P < 0.001; ****P < 0.0001. Nuclei: DAPI or TO-PRO-3. In the combo plot, the box represents means ± SD, the line in the box represents median, the lower and upper lines display the minimum and the maximum of the values, and the line on the right side of the box represents the sample distribution. Scale bars, (A) tile scans of spleen and kidney, 400 μm; for thymus and muscle, 200 μm; 50 μm for insets; (B) 20 μm.

Next, we extensively imaged and compared young and aging organs. We performed the analysis of images for regional differences, expression of cell and matrix markers, blood vessel distributions, blood vessel density, capillary diameters, artery diameters, and artery numbers (Fig. 2 and figs. S1, F to H, and S2 to S5). Hematoxylin and eosin (H&E) staining confirmed the healthy status of the aged tissues (fig. S2A). This analysis revealed that numerous organs exhibited a remarkable age-related loss in vascular abundance (Fig. 2A and fig. S2, B to D). The arterial numbers as determined by α-SMA staining declined in the aging murine organs including kidney, spleen, thymus, liver, and heart (Fig. 2A and fig. S2, B and D). In addition, the microvascular density declined in kidney, muscle, spleen, thymus, liver, and brain upon aging (Fig. 2A and fig. S2B). Moreover, our analysis of numerous EC markers confirmed that the decline in vascular density was not an artifact of a decline in the expression of certain cell surface markers. In addition to the decline of vascular density, another remarkable feature was the loss of PDGFRβ^+^ pericytes in these organs (Fig. 2B and fig. S2C). Investigation of vascular changes across different tissues illustrated further tissue-specific age-dependent changes in cell and matrix markers. For example, both thymus and spleen demonstrated increased Desmin-expressing cells upon aging (figs. S1G, S4, C and D, and S5B). A fundamental architectural change in kidney involved decreased renal corpuscle numbers and an increased renal corpuscle size upon aging (fig. S5A). In contrast, of the organs assessed, tissues with high remodeling capacity such as skin, gut, uterus, and lung exhibited no apparent changes in vessel density upon aging (Fig. 2C and fig. S5K). Thus, 9 (kidney, muscle, spleen, thymus, heart, liver, brain, bladder, and lung) of 12 organs demonstrated an age-dependent vascular attrition, while the remaining organs—gut, skin, and uterus—showed no age-related vascular changes (Fig. 2D and figs. S2, B to D, and S5K). Kidney, muscle, spleen, and thymus showed the most prominent changes in both vessel density loss and pericyte loss. Therefore, for the subsequent molecular and functional analysis underlying such age-dependent vascular attrition, we focused on this core set of three to four organs.

**Fig. 3:**
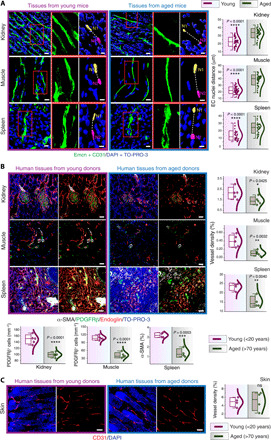
Age-related vascular changes in mouse and human tissues. (**A**) 3D images (left) show blood vessels in young (6-week-old) and aged (60-week-old) mouse kidney, muscle, and spleen. Combo plots (right) show quantification of EC nuclear distance in the young and aged organs (kidney, *n* = 42 from seven biological replicates; muscle, *n* = 47 from eight biological replicates; spleen, *n* = 37 from seven biological replicates). (**B**) Representative images of young and aged human tissue stained with α-SMA, PDGFRβ, and Endoglin. Combo plots (right) show a significant decline in vessel density in the kidney (*n* = 5), muscle (*n* = 5), and spleen (*n* = 4) upon aging. Combo plots (lower left) show quantifications of PDGFRβ^+^ cells in young and aged human kidney and muscle (*n* = 5). Combo plot (lower right) shows the quantification of α-SMA coverage (%) in young and aged human spleen (*n* = 4). (**C**) Representative images of young and aged human skin tissue. Combo plot (right) shows the quantification of vessel density in young and aged skin (*n* = 4). Data represent means ± SD. The *P* value derived from two-tailed unpaired *t* tests is given for all graphs. *P < 0.05; **P < 0.01; ***P < 0.001; ****P < 0.0001. Nuclei: DAPI or TO-PRO-3. N, nuclei. Scale bars, (A) 20 μm and 10 μm for insets; (B and C) 40 μm.

Further, the vascular loss was observed despite the age-dependent elongation and increase in the size of ECs in the aging tissues, as determined by distance analysis between nuclei of adjacent ECs (Fig. 3A and fig. S5L). Analysis of EC proliferation by fluorescence-activated cell sorting (FACS) quantifications with a proliferation marker Ki67 demonstrated an age-dependent decline in proliferating ECs (fig. S6, A and B). Analysis of apoptotic cell death by imaging-based TUNEL assay (fig. S6C) indicated an increase in EC apoptosis in thymus and muscle but not in kidney and spleen tissues, suggesting the involvement of organ-specific mechanisms in the regulation of age-dependent vascular loss.

Vascular leakage was analyzed to assess the impact of loss of pericytes on vessel integrity. No signs of vascular leakage were observed in the tissues from aging 55-week-old mice (fig. S6D). To analyze the impact of age-dependent loss of vessel density on the oxygenation status of the tissues, we performed pimonidazole staining on the sections of these four organs—kidney, muscle, spleen, and thymus. While the aging muscle and spleen demonstrated increased hypoxia, thymus and kidney demonstrated no changes (fig. S6E). Thus, such impacts of vascular loss on hypoxia were organ specific. While there was an age-dependent loss in vascular abundance, analysis of lymphatic vessels with LYVE-1 and PROX-1 showed no changes in lymphatic vessel density (fig. S7, A to D). Since LYVE-1 is also expressed by macrophages, lymphatic vessels were distinguished on the basis of their tubular shape. Quantifications of macrophage numbers by CD68 or F4/80 immunostainings showed no alterations with age (fig. S7E).

To understand the pathophysiological relevance of age-related changes observed in murine tissues, we analyzed human tissue samples. Healthy human tissues of young (<20 years) and aging (>70 years) individuals (table S4) were analyzed to understand vascular changes by performing immunohistochemistry for endothelial and pericyte markers (Fig. 3B). Comparable to murine tissues, aging human kidney, muscle, and spleen exhibited a significant decline in vessel density and pericyte numbers (Fig. 3B), while, similar to mice, aging human skin retained the vasculature (Fig. 3C). Thus, highly remodeling tissues with high regeneration potential such as skin retain blood vessel density during aging while other tissues show age-dependent vascular attrition. Liver, which has relatively high regeneration potential compared to other organs such as kidney, demonstrated an age-dependent vascular decline in mouse (fig. S2, B and D); however, human liver retained vascular density upon aging (fig. S7F). Thus, the above data illustrated the organ-specific loss of vascular abundance as a key feature of aging murine and human tissues.

### Vascular attrition is a harbinger of cellular aging

**Fig. 4:**
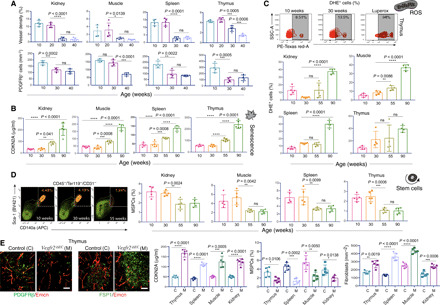
Vascular attrition precedes and dictates cellular aging. (**A**) Bar graphs of the kidney, muscle, spleen, and thymus for vessel density (top, *n* = 5) and PDGFRβ^+^ cells (bottom, *n* = 4 or 5). (**B**) Bar graphs show concentration of CDKN2A measured by ELISA in tissues from *p16LUC* mice aged 10, 30, 55, and 90 weeks (*n* = 5). (**C**) Representative FACS plots of side scatter area (SSC-A) versus PE-Texas red-A (representing DHE^+^ cells) in the thymus at 10 and 30 weeks and the positive control (Luperox treated). Bar graphs showing the quantification of DHE^+^ cells in tissues at different ages (kidney and spleen, *n* = 5; others, *n* = 4). (**D**) Representative FACS plots of MSPCs of thymus at 10, 30, and 55 weeks. Bar graphs showing quantification of MSPCs (%) in tissues at different ages (*n* = 5). (**E**) 3D images of thymus from *Vegfr2*^iΔEC^ (M) and littermate control (C) mice immunostained as indicated. Bar graphs showing the quantifications of CDKN2A concentration, MSPCs (%), and fibroblast numbers (*n* = 5). Data represent means ± SD. The *P* value derived from two-tailed unpaired *t* tests is given for two groups and one-way ANOVA test with Tukey’s multiple comparisons test for more than two groups. *P < 0.05; **P < 0.01; ***P < 0.001; ****P < 0.0001. Scale bars, (E) 50 μm.

To interrogate whether age-dependent vascular changes observed in numerous organs were the consequence of the cellular deterioration with aging, we investigated the time point when these tissue-level vascular changes started to dominate in the mouse life span. Toward this, organs from mice at different age groups were analyzed. The decline, both in blood vessel density and PDGFRβ^+^ pericyte numbers, was already evident in 30-week-old mice (Fig. 4A). Accumulation of senescent cells is a feature of aging tissues (*19*), and cellular senescence is marked by stable cell cycle arrest due to gradual telomere shortening (*20*) and derepression of the *INK4/ARF* locus (*21*). Thus, we analyzed a member of the INK4 family, an established senescence marker, p16/CDKN2A, using CDKN2A luciferase reporter mice (*22*). Luciferase/CDKN2A immunostaining was negative on tissue sections from 30-week-old mice (fig. S7G); however, luciferase/CDKN2A expression was detected in aged tissues (fig. S7G). Similarly, enzyme-linked immunosorbent assay (ELISA) analysis for CDKN2A demonstrated increased CDKN2A levels in 55-week-old mice or in 90-week-old mice as compared to the 10- and 30-week-old mice (Fig. 4B).

Various data establish the role of reactive oxygen species (ROS) in aging, and aged cells typically demonstrate increased accumulation of ROS. This increased ROS production occurs due to mitochondrial dysfunction with aging, which, in turn, leads to more mitochondrial degeneration and global cellular damage (*23*). Therefore, we analyzed the cellular ROS in tissues from mice at different age groups (Fig. 4C). ROS were detected using fluorescent probe dihydroethidium (DHE), which becomes fluorescent after reaction with superoxide and hydrogen peroxide in live cells (*11*). Flow cytometric analysis of DHE showed no significant increase in ROS accumulation in tissues from 30-week-old mice compared to the 10-week-old mice (Fig. 4C). Luperox, an organic peroxide, was used as a positive control.

The decline in the regenerative capacity of organs is an apparent feature of mammalian aging caused by the functional decline of adult stem cells in multiple tissues due to their deficient proliferation (*24*). To analyze the status of stem cells in mice with different age groups, we analyzed and quantified mesenchymal stem/progenitor cells (MSPCs) as they reside in numerous tissues and have a perivascular location (*25*). Flow cytometric analysis of MSPCs as CD45^−^/Ter119^−^/CD31^−^/Sca-1^+^/CD140a^+^ (*26*) showed no significant differences in MSPC frequency between 10- and 30-week-old mice, while the 55- and 90-week-old mice showed a decline in MSPCs (Fig. 4D). Another feature of aging is the presence of chronic low-grade inflammation, frequently associated with the increase in interleukin-6 (IL-6) and IL-1β levels (*27*). ELISA analysis for IL-6 demonstrated increased IL-6 levels in aging 55-week-old tissues but not as early as 30-week-old tissues when the vascular changes emerge (fig. S7H). The above results demonstrated that vascular and pericyte loss occurred early in the mouse life span and preceded the cellular hallmarks of aging. The aging and stress-related tissue-specific vascular and other cellular changes are provided in table S5. Therefore, the observed age-dependent vascular attrition was not a consequence of aging-associated cellular deterioration.

Next, we examined whether the vascular alterations affect cellular hallmarks of aging. VEGFA-VEGFR2 signaling is a critical driver of physiological and pathological neoangiogenesis, and loss of VEGFR2 on ECs inhibits angiogenesis and vascular remodeling (*28*). Therefore, we examined the effect of interfering with VEGFA-VEGFR2 signaling. Specifically, we generated EC-specific loss-of-function mice (*Vegfr2*^iΔEC^) by combining loxP-flanked Vegfr2 alleles (*Vegfr2*^loxP/loxP^) and Cdh5(PAC)-CreERT2 transgenics. Following tamoxifen administration in the adult 8-week-old mice, analysis of kidney, muscle, spleen, and thymus tissues from *Vegfr2*^iΔEC^ mice was performed at the age of 11 weeks. The deletion efficiency was confirmed by quantitative polymerase chain reaction (qPCR) for *Vegfr2* (fig. S7I). The tissues from *Vegfr2*^iΔEC^ mutant mice demonstrated loss of blood vessels and PDGFRβ-expressing pericytes (Fig. 4E and fig. S7J). Increased CDKN2A levels were detected in mutant mice as compared to their littermate control mice (Fig. 4E). These results indicated that an accumulation of senescent cells was associated with vascular loss. However, CDKN2A levels remained unchanged in sorted ECs from *Vegfr2*^iΔEC^ mice compared to their littermate controls, indicating that while vascular loss is associated with the cellular senescence in the tissue microenvironment, ECs themselves are not triggered to senescence in these mice (fig. S7K). In addition, *Vegfr2*^iΔEC^ mutant mice exhibited a decline in MSPC numbers (Fig. 4E); however, there was an accumulation of fibroblasts (Fig. 4E). Thus, the above results demonstrated that vascular deterioration affected the cellular hallmarks of aging and led to global cellular deterioration.

### Age-dependent differentiation of pericytes into fibroblasts

**Fig. 5:**
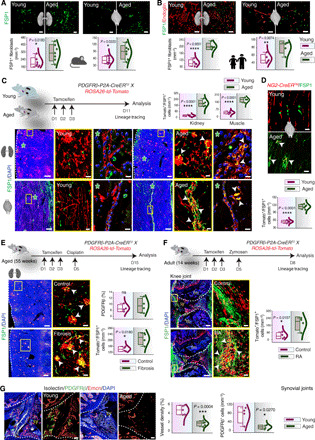
Pericytes to fibroblast differentiation during aging, fibrosis, and arthritis. (**A**) FSP1 staining and fibroblast numbers in murine tissues (*n* = 7). (**B**) FSP1 staining and fibroblast quantifications in human tissues (*n* = 5). (**C**) FSP1 immunostaining in *PDGFR*β*-P2A-CreERT2; ROSA26-td-Tomato* mice. Asterisks indicate higher magnifications of regions at single-cell resolution. Combo plots show tomato^+^/FSP1^+^ cell numbers (*n* = 4 or 5). (**D**) FSP1 staining and quantifications of tomato^+^/FSP1^+^ cell numbers in *NG2-CreERTM; ROSA26-td-Tomato* young and aged muscle (*n* = 5). (**E**) FSP1 staining in control and fibrosis kidney and quantifications of PDGFRβ coverage and tomato^+^/FSP1^+^ cell numbers (*n* = 4). (**F**) FSP1 immunostaining and quantifications on the synovial joints from *PDGFR*β*-P2A-CreERT2; ROSA26-td-Tomato* control and RA mice (*n* = 4). (**G**) 3D images of young and aged mouse joints immunostained as indicated. Combo plots show vessel density (*n* = 5) and PDGFRβ^+^ cell numbers (*n* = 4). Data represent means ± SD. The *P* value is derived from two-tailed unpaired *t* tests. *P < 0.05; **P < 0.01; ***P < 0.001; ****P < 0.0001. Yellow boxes show insets at higher magnification. Arrowheads represent tomato^+^/FSP1^+^ cells. fm, femur; tb, tibia; sy, synovia. Nuclei: DAPI. Scale bars, (A and B) 40 μm; (D) 50 μm; (C, E, and F) tile scans, 250 μm; insets, 50 μm; (G) 50 μm.

Analysis of vascular microenvironments across mouse organs demonstrated increased immunostaining for fibroblast markers upon aging, namely Podoplanin and FSP1 (*29*–*31*) (Fig. 5A and fig. S8A). In addition, the *Vegfr2*^iΔEC^ mutant mice demonstrated significantly increased fibroblast numbers (Fig. 4E). Further, quantification of fibroblasts demonstrated a significant increase in FSP1-positive fibroblasts in the aging tissues (Fig. 5A). Similarly, analysis and quantification of fibroblast numbers, as determined in human tissues based on FSP1 expression, demonstrated increased fibroblasts in tissues from aging donors compared to young donors (Fig. 5B). Moreover, the overlapping expression of FSP1 and Decorin, an angiogenesis-inhibiting matrix proteoglycan, was increased in aging tissues (fig. S8B).

The aging tissues exhibited an expansion of fibroblasts (Fig. 5, A and B) but a decline of pericytes (Figs. 2B and 4A); therefore, we speculated that the age-dependent loss of pericytes is due to their differentiation into fibroblasts. To examine this, we performed genetic lineage tracing of pericytes in young and aging mice, as it is a powerful approach for the investigation of cell fate and differentiation (*32*). Tamoxifen injections were performed in young (6-week-old) and aging (55-week-old) *PDGFRb-CreERT2* x *ROSA26 td-Tomato* double transgenics (Fig. 5C). The analysis of mice at day 11 after tamoxifen administration led to tomato expression in pericytes in both young and aging mice and, as expected, with a decline in aging mice (Fig. 5C and fig. S8C). Immunostaining for FSP1 showed rare or no overlap between tomato-positive pericytes and FSP1 expression in the young mice (Fig. 5C); however, the aging mice demonstrated abundant double-positive cells with tomato and FSP1 expression (Fig. 5C). Thus, pericytes were precursors for and generated FSP1-expressing fibroblasts in aging mice but not in young mice. We further tested this hypothesis using another pericyte-specific inducible transgenics, *NG2-CreERTM* × *ROSA26td-Tomato*. Again, at day 11 after tamoxifen administration, FSP1 expression was rarely detected in tomato-positive cells in young mice (Fig. 5D), but double-positive (tomato- and FSP1-expressing) cells were abundant in the aging mice (Fig. 5D).

### Pericyte-derived fibroblasts contribute to organ fibrosis during aging and in arthritis

Nonhematopoietic, tissue-resident fibroblasts contribute to the pathogenesis of several diseases (*33*) and injury-induced fibrosis (*34*). Notably, FSP1 expression promotes fibrosis, and FSP1-positive fibroblasts have been shown to drive fibrosis (*31*). Therefore, to investigate the involvement of pericytes during fibrosis, we next analyzed the fate of pericytes in young and aging mice during the injury-induced fibrosis in the kidney. *PDGFRb-CreERT2* × *ROSA26 td-Tomato* mice were injected with cisplatin to induce kidney fibrosis, and lineage tracing was performed (Fig. 5E and fig. S8D). H&E staining was used to display the healthy status of the cisplatin-treated mice (fig. S8E). The lineage tracing of the kidney tissues from control and cisplatin-treated mice demonstrated a significant increase in the tomato and FSP1 double-positive cells compared to tissues from the littermate control mice (Fig. 5E and fig. S8D). In addition to FSP1 expression, a fraction of these tomato-positive fibroblasts also expressed Podoplanin, Vimentin, and PDGFRα (fig. S8F). Further, the frequency of these tomato and FSP1 double-positive cells was significantly higher in the aging mice developing fibrosis compared to the young mice with fibrosis (fig. S8G). Thus, differentiation of pericytes to fibroblasts is augmented in injury-induced fibrosis occurring during aging.

In addition to fibrosis, fibroblasts contribute to the pathogenesis of inflammatory diseases (*35*), and synovial fibroblasts, in particular, contribute to the joint damage and inflammation in rheumatoid arthritis (RA) by taking on invasive, tissue-destructive phenotype (*33*, *36*). Therefore, we next investigated whether pericyte-derived fibroblasts contribute to fibroblast proliferation and expansion in arthritis. Thus, we used an experimental model of RA; zymosan was injected into the knee joints of *PDGFRb-CreERT2* × *ROSA26 td-Tomato* mice to induce arthritis (*37*) with the aim of tracking the pericytes in synovial joints. Lineage tracing in this mouse line demonstrated significantly increased tomato and FSP1 double-positive cells in the arthritic joints but not in control joints (Fig. 5F). Thus, pericytes were a source of fibroblasts in experimental inflammatory arthritis. Further, a comparison of young versus aging synovial joint tissues demonstrated an increase in fibroblasts with a decline in pericytes and blood vessels in aging mice as compared to young mice (Fig. 5G and fig. S8H). Together, the above data indicated that pericyte-fibroblast differentiation was a prominent feature of two inflammatory disease states, in which fibroblasts contribute in different ways.

### Down-regulation of vascular and pericyte maintenance pathways in aging ECs

**Fig. 6:**
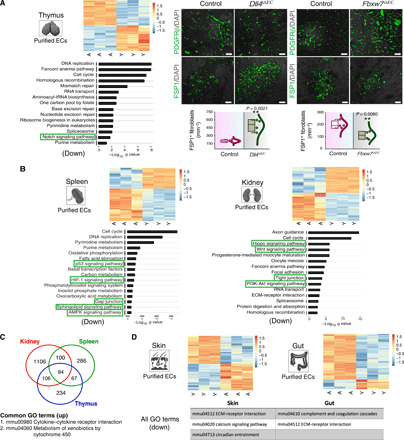
Molecular changes in the aging endothelium. (**A**) Heatmap (left) shows RNA-seq expression levels of differentially expressed genes between young (Y; 8- to 10-week-old) and aged (A; 50- to 55-week-old) thymus (FDR-adjusted *P* value cutoff of <0.01). The color intensity represents the row scaled normalized log_2_(CPM) expression, whereas the column represents the replicates. Red and blue color intensity indicates up-regulated and down-regulated genes. Bottom left showing the most significant down-regulated biological processes obtained from gene set enrichment. 3D images show PDGFRβ and FSP1 immunostaining in *Dll4*^iΔEC^ and littermate control thymus as well as *Fbxw7*^iΔEC^ and control thymus (11-week-old). Combo plots show fibroblast numbers (*n* = 4). (**B**) Heatmaps showing RNA-seq expression levels of differentially expressed genes between young (Y; 8- to 10-week-old) and aged (A; 50- to 55-week-old) spleen and kidney. Bottom shows the most significant GO terms of the down-regulated pathways. (**C**) Venn diagram of biological processes obtained from gene set enrichment analysis representing the up-regulated pathways from kidney, spleen, and thymus. (**D**) Heatmaps showing RNA-seq expression levels of differentially expressed genes between young (Y) and aged (A) skin and gut. Bottom shows the GO terms of the down-regulated pathways. Data represent means ± SD. The *P* value is derived from two-tailed unpaired *t* tests. **P < 0.01. Nuclei: DAPI. Scale bars, (A) 50 μm. ECM, extracellular matrix.

To delineate the molecular mediators driving age-associated changes and vascular loss, RNA sequencing (RNA-seq) of purified young versus aging ECs from thymic tissues was performed (Fig. 6A). Thymus tissue was analyzed here, as it is known to undergo remarkable changes with aging. Young and aging mouse thymi were processed to obtain single-cell suspensions. ECs were isolated from these organs by a magnetic bead–based separation method using the anti-Endomucin antibody and subjected to RNA-seq analysis. Unsupervised clustering analysis indicated distinct profiles of ECs between young and aging mice (Fig. 6A). Differentially regulated mRNAs were identified on ECs from aging mice with EC from young mice as reference samples. Significantly differentially expressed genes were obtained with a cutoff false discovery rate (FDR)–adjusted *P* value of <0.01; 491 genes in the thymus were up-regulated, while 503 were down-regulated (Fig. 6A). We performed gene set enrichment for pathway analysis (GAGE) using the KEGG (Kyoto Encyclopedia of Genes and Genomes) database. The significant pathways were filtered with a *q* value of <0.1. The aging ECs showed down-regulation of Notch signaling (Fig. 6A). Notch and its ligand Dll4 regulate angiogenesis in a tissue-specific manner. In several tissues, Notch is a negative regulator of angiogenesis, but in the skeletal system, it is a positive regulator of angiogenesis (*38*). Therefore, we next performed inducible targeting of the *Dll4* gene. Analysis of the thymic tissues in *Dll4*^iΔEC^ mutants (11-week-old) demonstrated loss of blood vessels, a decline in pericytes, and an increase in fibroblasts (Fig. 6A and fig. S9A). In contrast to these, the EC-specific Notch gain-of-function *Fbxw7*^*i*ΔEC^ mice (11-week-old) demonstrated an increase in blood vessels and pericytes and a decline in fibroblast numbers (Fig. 6A and fig. S9A). These results suggested that age-dependent loss of Notch signaling drove global vascular and perivascular aberrations. Furthermore, the vascular changes in *Dll4*^iΔEC^ mutants led to a massive reduction in T cell progenitors and perturbed development of thymic epithelium, thereby negatively affecting the thymus microenvironment and function (fig. S9, B to E). These findings suggested that age-dependent vascular loss was causally linked to decreased thymic output of mature T cells (*39*, *40*).

Next, to investigate whether this down-regulation of the endothelial Notch signaling with aging is a common feature across multiple tissues, we performed RNA-seq analysis of the ECs from spleen and kidney tissues derived from young (8- to 10-week-old) and aging (50- to 55-week-old) mice. A similar analytical approach, as described above for the thymus, was followed. A total of 1397 genes in the kidney and 537 in the spleen were up-regulated, while 1286 genes in the kidney and 341 in the spleen were down-regulated upon aging (Fig. 6B and fig. S10, A and B). As before, we performed GAGE using the KEGG database in these aging ECs. The significant pathways were filtered with a *q* value of <0.1. The top down-regulated pathways in both of these organs showed down-regulation of the pathways known to be critical for vascular and pericyte maintenance and remodeling (Fig. 6B). However, the Notch signaling was not altered upon aging in these tissues (Fig. 6B). Differential gene expression in the kidney showed a down-regulation of Hippo and Wnt signaling pathways, which are required for angiogenesis and blood vessel maintenance (Fig. 6B) (*41*, *42*). In the spleen, aging ECs demonstrated down-regulation of metabolic pathways, including HIF1α signaling (Fig. 6B) (*43*), and also, gap junction communication (*44*) was down-regulated (Fig. 6B). These findings suggested that multiple age-dependent alterations in known vascular maintenance pathways converge on the loss of vasculature in different tissues. We further interrogated the common EC aging signature among these three tissues analyzed: thymus, spleen, and kidney. Aging ECs from these tissues demonstrated 84 common up-regulated genes and identified up-regulation of cytokine–cytokine receptor interaction and metabolism of xenobiotics by cytochrome 450 by GAGE analysis, indicating the proinflammatory nature of the aging ECs among these tissues (Fig. 6C and fig. S10, C to E). The GO (Gene Ontology) terms of the up-regulated and down-regulated pathway of kidney, spleen, and thymus are provided in table S6. We then compared the young and aging ECs from gut and skin, as these tissues retained vascular density upon aging. Young and aging mouse gut and skin were processed to obtain single-cell suspensions and subjected to RNA-seq analysis as performed for the thymus, spleen, and kidney. No common up-regulated or down-regulated genes and pathways were identified between all the five tissues gut, skin, thymus, spleen, and kidney (Fig. 6D and fig. S10F). Unlike those from the thymus, spleen, and kidney, aging ECs from the gut and skin lacked up-regulation of cytokine–cytokine receptor interaction pathway, indicating the lack of proinflammatory nature of these ECs (tables S7 and S8). Thus, the major distinguishing factor between the ECs from the tissues demonstrating the loss of vasculature versus tissues retaining vasculature was up-regulation of the inflammatory processes in the ECs derived from the tissues with vascular loss. These results suggest that ECs in highly remodeling tissues that retain vessel and pericyte abundances may have evolved with protective mechanisms to shield from inflamm-aging and associated inflammatory damage occurring during aging.

## DISCUSSION

The identification and regulation of signals driving the aging process are long-standing goals in physiology. Aging negatively affects organ function (*45*). The description of the tissue-level age-associated changes in the literature remains restricted to the gross structural and tissue changes such as the increase in tissue stiffness and adiposity (*15*, *27*, *39*, *40*, *46*). In this study, we implement a large-scale 3D spatial comparison of vascular cells and molecules in young and aging mouse tissues from several organs to define the major changes across both axes. This in-depth analysis of aging tissues revealed vascular attrition as a primary hallmark of aging and provides unprecedented insights into the microenvironmental tissue-level changes during aging. Further, we make the 3D tissue maps available freely as an image database. These more than 1000 single-cell–resolution 3D maps with spatial information are available for further exploration and quantitative analyses. This resource will serve as an essential research tool to understand tissue biology in various fields of physiology, aging, matrix, and vascular biology and to investigate functional pathophysiology and therapeutic effects.

Our imaging datasets reveal that the loss of vascular abundance accompanied by the decline in pericytes is a key feature of aging tissues. This is the first comprehensive study highlighting age-dependent vascular changes across several organs. Loss of vessel density and pericytes emerges as the mark of aging organs and tissues; however, highly remodeling tissues with high regeneration potential, such as the skin, gut, and uterus (*47*–*49*), preserve the abundance of the blood vessels and pericytes with aging. Similarly, vessel densities remain unaffected in aging bones (*14*), which have relatively higher regeneration potential compared to tissues such as the kidney, spleen, heart, or brain. Thus, stage and extent of vascular attrition are likely to direct the regenerative limitations of a tissue. Further, observations described herein corroborate the findings in injury-induced organ fibrosis where pericytes differentiate into fibroblasts to drive fibrosis. Our findings also demonstrate that pericytes are a source of fibroblasts in joint inflammation and that the differentiation of pericytes to fibroblasts increases with aging. This is of particular significance, given the importance of fibroblasts as a source of inflammatory mediators in RA. Last, EC-specific genetic manipulations prove that vascular loss drives cellular changes such as senescence. Together, these findings imply that the strategies to inhibit age-dependent changes in vasculature such as the loss of vascular abundance and pericyte to fibroblast differentiation have the potential to delay or even prevent cellular dysfunction during aging.

Perivascular cells are known to contribute to diseases such as fibrosis (*50*), and pericyte to fibroblast transition is reported to occur in tumors and promote tumor growth and metastasis (*51*). Fibroblasts are known to support the growth of cancer cells (*52*, *53*). Our results attest pericyte to fibroblast differentiation during aging and pericytes as a source of fibroblasts during aging. Molecular analysis of aging endothelium across three different tissues demonstrates the down-regulation of multiple signaling pathways regulating blood vessel maintenance and remodeling. However, the accumulation of these alterations with aging is heterogeneous across the tissues, nonetheless all leading to a common path of vascular attrition and proinflammatory nature of ECs. We propose that EC inflammation along with diverse genes and signals drive the vascular and perivascular alterations with age, ultimately leading to vascular loss and fibroblast accumulation. Investigation and dissection of tissue-specific mechanisms regulating perturbations of the molecular cross-talk between the EC and neighboring tissue cells during aging will provide further insights into the vascular control of the aging process.

## MATERIALS AND METHODS

### Mice

C57BL/6J mice were purchased from the Jackson laboratory and bred in our facilities. Both male and female mice were included in all the experiments and analyses. Mice of 6-week-old and 56- to 70-week-old age were selected for the initial analysis of young versus aging vascular and perivascular changes. Further, analysis and quantifications were validated across age groups of 10, 20, 30, 40, 55, and 90 weeks, unless otherwise mentioned in the figure panels or legends. For all the disease models and transgenic mice, 11- to 15-week-old young mice or 55-week-old aging mice were used, unless otherwise stated. For some of the imaging analysis to test and validate different antibodies, tissues were also harvested from 2- to 4-week-old mice. For senescence analysis, the *p16INK4a*-Luciferase (*p16LUC*) model was used (*22*). *PDFGRb-P2A-CreERT2* (stock no. 030201), *NG2-CreERTM* (stock no. 008538), *ROSA26 td-Tomato* (stock no. 007914), and *Vegfr2*^loxP/loxP^ (stock no. 018976) mice were purchased from the Jackson laboratory. For genetic labeling, *PDFGRb-P2A-CreERT2* and *NG2-CreERTM* transgenic mice were mated with *ROSA26 td-Tomato* reporters. In addition, inducible EC-specific *Vegfr2* knockout mice (*Vegfr2*^iΔEC^) were generated. Briefly, transgenic *Vegfr2*^loxP/loxP^ mice were bred with *Cdh5(PAC)-CreERT2* transgenic mice (*9*), and Cre-negative *Vegfr2*^loxP/loxP^ mice (control) and Cre-positive *Vegfr2*^loxP/loxP^ (*Vegfr2*^iΔEC^) mutant mice were used in this study. To investigate the Notch signaling pathway in the thymus, transgenic *Dll4*^loxP/loxP^ mice were bred with *Cdh5(PAC)-CreERT2* transgenic mice to generate EC-specific *Dll4* knockout mice (*Dll4* ^iΔEC^) as described previously (*38*). *Fbxw7*^lox/lox^ mice were bred with *Cdh5(PAC)-CreERT2* transgenic mice to generate EC-specific *Fbxw7* knockout (*Fbxw7*^iΔEC^) mice as described previously (*9*, *38*). All animals were genotyped by PCR.

All animal experiments were conducted under the Home Office Guidance on the Operation of the Animals (Scientific Procedures) Act 1986. All animals received humane care according to the “Principles of Laboratory Animal Care” formulated by the National Society for Medical Research and the guide for the Care and Use of Laboratory Animals (National Academies Press, 2011). All animal experimentation at the University of Oxford was approved by the local Animal Welfare and Ethical Review Board and by the U.K. Government Home Office (Animals Scientific Procedures Group).

### Human samples

Formalin-fixed paraffin-embedded (FFPE) standard tissue blocks (kidney, muscle, spleen, and liver) were obtained from Amsbio (Oxford, UK). Human skin samples were obtained from West China Hospital, Sichuan University. Samples from young donors (<20 years old) and old donors (>70 years old) were selected for the comparison analysis between the young and aged groups, respectively. Human specimens provided by AMS Biotechnology (Europe) Limited and West China Hospital are legally procured under the laws and regulations of the country. All samples are collected by AMS Biotechnology from consented patients in collaboration with major research/clinical centers under the local Ethics Committee/Institutional Review Board–approved protocols. Histological study was performed to confirm that sample tissues were healthy and lacked disease components. Sample tissues were confirmed of any pathological features by histological study, and details of healthy young and aged human samples are provided in table S4.

### Tamoxifen treatment for inducible gene deletion

For oral gavage of tamoxifen, tamoxifen (Sigma-Aldrich, T5648) was first dissolved in 100% ethanol and then suspended in corn oil to a final concentration of 5 mg/ml. For *Vegfr2*^iΔEC^ mutant mice, to induce Cre activity and gene inactivation, *Vegfr2*^iΔEC^ mutant mice and control littermate mice were orally treated with tamoxifen at a dose of 50 mg/kg of body weight for three consecutive days. Mice were euthanized and analyzed 2 weeks after the last dose of tamoxifen. For *Dll4* ^iΔEC^ and *Fbxw7*^iΔEC^ mutant mice, to specifically delete endothelial *Dll4* and *Fbxw7*, *Cdh5(PAC)-Cre* activity was induced by a tamoxifen dose of 50 mg/kg of body weight provided orally in three consecutive days. Eleven-week-old *Dll4* ^iΔEC^ and *Fbxw7*^iΔEC^ mutant mice and the control littermate mice were euthanized and analyzed 2 weeks after the last dose of tamoxifen.

### Genetic lineage tracing

For tamoxifen-induced genetic lineage tracing, *PDFGRb-P2A-CreERT2* × *ROSA26 td-Tomato* and *NG2-CreERTM* × *ROSA26 td-Tomato* transgenic mice were used. To induce Cre activity, tamoxifen was treated orally at a dose of 50 mg/kg for three consecutive days. At the indicated time, mice were euthanized by CO_2_ asphyxiation, and organs were collected for analysis.

### Cisplatin-induced fibrosis

*PDFGRb-P2A-CreERT2* × *ROSA26 td-Tomato* transgenic mice were used for the cisplatin-induced fibrosis experiment. To induce Cre activity, aged transgenic mice (55-week-old) and young transgenic mice (8-week-old) were first orally treated with tamoxifen at a dose of 50 mg/kg of body weight for three consecutive days. Two days after the last dose of tamoxifen, to induce fibrosis, cisplatin (Cayman Chemical, 13119) at a dose of 20 mg/kg of body weight in phosphate-buffered saline (PBS) was administered by intraperitoneal injection, and the control littermate mice were injected with an equivalent volume of physiological saline. Organs were collected for analysis 10 days after cisplatin administration. Cisplatin treatment is 90% lethal after 2 weeks; therefore, mice were weighed and carefully monitored for any signs of infection.

### Zymosan-induced arthritis

*PDFGRb-P2A-CreERT2* × *ROSA26 td-Tomato* transgenic mice were used for the zymosan-induced arthritis experiment. To induce Cre activity, adult transgenic mice (14-week-old) were first orally treated with tamoxifen at a dose of 50 mg/kg of body weight for three consecutive days. Two days after the last dose of tamoxifen, to induce arthritis, mice received an intra-articular injection of zymosan (180 μg) (Sigma-Aldrich, Z4250) into one knee joint, and the contralateral knee was injected with the same volume of saline as control. Joints with both femur and tibia were collected for analysis after 48 hours after injection.

### Metabolic labeling with hypoxia probe

For metabolic labeling with the hypoxia probe pimonidazole (Hypoxyprobe Inc., hp15-100kit), young and aged C57BL/6J mice were intraperitoneally injected with pimonidazole (60 mg/kg). One hour after the injections, mice were euthanized. Then, organs were collected from young and aged mice. Metabolized pimonidazole was detected by a rabbit antiserum against the nonoxidized, protein-conjugated form of pimonidazole (Hypoxyprobe).

### Histology

Mice were euthanized, and organs were dissected, collected, photographed, and fixed with 10% formalin in PBS. Samples were embedded in a paraffin block and sliced into 5-μm-thick sections. Following H&E staining, the samples were imaged by bright-field microscopy (Olympus Co., Japan).

### Sample preparation for immunostaining

The samples of murine organs were freshly dissected from C57BL/6J mice and fixed in ice-cold 2% paraformaldehyde solution for 4 hours. Next, the samples were thoroughly washed with PBS and kept for 12 hours in a solution containing 20% sucrose (Sigma-Aldrich, S9378) and 2% polyvinylpyrrolidone (PVP; Sigma-Aldrich, PVP360) solution at 4°C. Samples were then incubated in ice-cold solution containing 30% polyethylene glycol and 0.5% Triton X-100. Before cryosectioning, the tissues were embedded in 8% gelatin (Sigma-Aldrich, G2625) solution supplemented with 20% sucrose and 2% PVP. Samples were then sectioned at 100 to 150 μm thickness by a Leica CM3050 cryostat with low-profile blades (Leica, 14035838382) and allowed to air-dry.

For preparation of bone tissues, freshly dissected femur and tibia with the joint were fixed in ice-cold 4% paraformaldehyde solution for 4 hours. Decalcification was conducted with 0.5 M EDTA with shaking at 4°C for 48 hours, and decalcified bones were then immersed into 20% sucrose and 2% PVP solution for another 24 hours. Bone tissues were embedded in an 8% gelatin solution supplemented with 20% sucrose and 2% PVP. Samples were sectioned by a Leica CM3050 cryostat with low-profile blades (Leica, 14035838382) and allowed to air-dry. For FFPE human tissue blocks, after the tissue blocks were frozen at −20°C for 30 min, 12-μm sections were prepared using a Leica RM2235 manual rotary microtome and microtome blades (FEATHER, 207500006).

### Immunostaining

For immunostaining analyses, tissue sections were air-dried for 15 min and hydrated with PBS. After permeabilization in 0.3% Triton X-100 for 10 min and blocking in 5% donkey serum at room temperature (RT), samples were incubated with primary antibodies diluted in blocking buffer (1:150) overnight at 4°C or for 4.5 hours at RT. Following seven washes (each for 3 min) in PBS solution, sections were then incubated with Alexa Fluor–conjugated antibodies (1:300) for 1.5 hours at RT, washed five times further, and flat-mounted on microscope glass slides with Fluoromount-G (Invitrogen, 00-4958-02). Nuclei were counterstained with TO-PRO-3 or 4′,6-diamidino-2-phenylindole (DAPI). For negative controls, immunostaining with no primary antibodies was performed.

Primary antibodies used are provided in table S1. Secondary antibodies used are as follows: donkey anti-rat immunoglobulin G (IgG) Alexa Fluor 594 (A-21209, Thermo Fisher Scientific), donkey anti-goat IgG Alexa Fluor 488 (A-11055, Thermo Fisher Scientific), donkey anti-goat IgG Alexa Fluor 647 (A-21447, Thermo Fisher Scientific), donkey anti-goat IgG Alexa Fluor 546 (A-11056, Thermo Fisher Scientific), Alexa Fluor 488 streptavidin conjugate (S11223, Thermo Fisher Scientific), Alexa Fluor 546 streptavidin conjugate (S11225, Thermo Fisher Scientific), donkey anti-rabbit IgG Alexa Fluor 488 (A-21206, Thermo Fisher Scientific), and donkey anti-rabbit IgG Alexa Fluor 647 (A-31573, Thermo Fisher Scientific).

### Immunohistochemistry on paraffin-embedded human samples

Human tissue sections generated from the FFPE blocks were deparaffinized in xylene for two times (each for 3 min) and xylene/ethanol (1:1) solution for 3 min at RT. After dehydration through graded ethanol solutions and rinsing in cold water, sections were incubated in preheated heat-induced antigen retrieval using a universal heat-induced epitope retrieval (HIER) solution (Abcam, ab208572) at 95°C for 10 min and washed thoroughly in distilled water. After air-drying, sections were processed for immunostaining, as described above.

### Imaging setup and image acquisition

3D immunofluorescent images for organs were captured at single-cell resolution on a laser scanning confocal microscope. The imaging equipment setup consisted of a Zeiss laser scanning microscope 880 equipped with seven laser lines (405, 453, 488, 514, 561, 594, and 633 nm), Axio Examiner (upright) stand and Colibri 7 epifluorescence light source with LED (light-emitting diode) illumination, four objectives, fast scanning stage with PIEZO XY, 32-channel gallium arsenide phosphide detector (GaAsP) PMT (photomultiplier tube) plus two-channel standard PMT, acquisition, and analysis software including measurement, multichannel, panorama, manual extended focus, image analysis, time lapse, Z Stack, extended focus, autofocus, and with additional modules: Experiment Designer and Tiles and Position. A 20× Plan Apo/0.8 dry lens, 20× Plan Apo 1.0 DIC VIS-IR D0.17 water dipping lens, and 10× Plan Apo 0.45 WD = 2.0 M27 dry lens were used for tiling. Large regions through the thick sections of organs were imaged using the tile scan function with appropriate numbers of tiles according to the specific size of each sample, and images were then stitched with a 10% overlap using Zen Black (version 3.1, Zeiss) software. All instrument settings were kept the same between acquisitions of young and aged samples for each organ or between littermate controls and mutants. Z-stacks of images were processed and reconstructed in three dimensions with Imaris software (version 9.5.0, Bitplane). Imaris, Adobe Photoshop, and Adobe Illustrator software were used for image processing and analysis in line with the *Science Advances* guidance for image processing.

### 3D surface reconstruction

3D surface rendering in images was applied using the surface module in Imaris. Briefly, the ROI was defined, and a single channel of blood vessels, perivascular cells, or matrix markers was reconstructed with surface segmentation. Followed by smoothness of the ROI, the background subtraction option was used for the threshold settings. After the threshold adjustment, the manual option was set up to reach a proper value according to the preview. Last, the resulting images were visually inspected to manually remove small individual segmented components of high sphericity, which were regarded as noise.

### EC apoptosis assay

For detection of EC apoptosis in young and aged tissues, DNA fragmentation (TUNEL) imaging assay kit (Abcam, ab66110) was used according to the manufacturer’s instructions. Briefly, frozen tissue sections were immunostained with Emcn then washed with PBS, and proteinase K solution was added. Next, sections were rinsed with DNA labeling solution, followed by adding the antibody solution and the 7-AAD/ribonuclease solution. After thoroughly rinsing, sections were sealed, and images were acquired using confocal microscopy.

### Quantifications of imaging datasets

Vascular quantifications such as vessel density, capillary diameter, artery number, and artery diameter were conducted using Imaris (version 9.5.0) or Fiji (version 2.0.0) based on single-cell–resolution 3D images. Most of the results of the quantification obtained on one software were confirmed on the other to guarantee the findings; thus, two softwares were used for image analysis. Quantifications of vessel density and artery numbers were performed across the entire thick longitudinal sections of the organs to avoid regional bias. For the analysis of vessel density, the Imaris Surface Analysis XTensions tool was used. Briefly, using the Crop 3D tool, the total tissue volume was acquired via the Volume Statistics function in Imaris. Then, a single channel of an endothelial marker was reconstructed in 3D using the Surface function, and the tissue volume of blood vessels was measured using the Surface Statistics function. The vessel density was calculated by dividing the tissue volume of blood vessels in the numerator by the total tissue volume of the whole organ in the denominator. For the quantifications of artery numbers, a single channel of α-SMA (Sigma-Aldrich, C6198) was used. Arteries were then distinguished on the basis of their structure and tubular shape. The artery numbers per 1 mm^2^ of tissue area based on the nuclear stain (DAPI or TO-PRO-3) were calculated and graphed.

For the quantifications of capillary diameters, endothelial markers were used, and for artery diameter analysis, α-SMA immunostaining was used. Since α-SMA could also be expressed by some stromal cells, arteries were distinguished on the basis of their structure and tubular shape. Briefly, a single channel of capillaries or arteries was projected with max intensity mode in Fiji. Multiple random regions in each section of organs were selected for quantifications. The average diameter of capillaries and arteries in each sample was then calculated according to the measurements using the distance tools.

Analysis and quantification of EC nuclei distance in young and aged muscle and spleen were conducted with Fiji (version 2.0.0) and Imaris based on single-cell–resolution 3D images. Briefly, channels of cell surface endothelial marker (Emcn or CD31) and nuclear stain (DAPI or TO-PRO-3) were merged and projected with max intensity mode after the ROI was defined. According to the colocalization between an endothelial marker and nuclei staining, EC nuclei were selected, and the distance of the adjacent EC nuclei was measured by the Distance tool in Fiji. The adjacent EC nuclei were then surface rendered with different colors using the Surface module in Imaris.

F4/80 or CD68 immunostaining was used to identify and quantify macrophage numbers in young and aged organs. Random regions in each tissue section were selected from each sample, and the numbers of CD68- or F4/80-positive macrophages were recorded with the merge of the nuclear stain TO-PRO-3 channel and CD68 or F4/80 channel. Next, the number of CD68- or F4/80-positive macrophages per area (square millimeter) was calculated by dividing the number of labeled cells by the total area of the selected region.

For the analysis and quantification of cell death by TUNEL assay, nuclei detection and membrane detection function in the Cell module of Imaris were used to automatically segment and analyze the numbers of TUNEL^+^ ECs. Under the Cell Creation Wizard of Imaris, the Emcn channel was selected as source channel for membrane-based detection, and the Tunel channel was selected for nuclei-based detection. Nuclei locations were used as seed points for an algorithm performing a cell membrane calculation, which was used to distinguish between the inner and outer boundaries of the cell. The final results displayed accurate segmentation of cells and presented the total number of TUNEL^+^ ECs. Next, the number of TUNEL^+^ ECs per tissue area (square millimeter) was calculated by dividing the number of segmented cells in the numerator by the total tissue volume in the denominator.

Quantification of cell numbers such as PDGFRβ^+^ pericytes or FSP1-positive fibroblasts was conducted with Fiji (version 2.0.0) based on single-cell–resolution 3D images. The quantification findings and trend of changes were also confirmed by using the Imaris software. Quantifications were performed on numerous thick sections spanning each organ and across multiple biological replicates as indicated in the figure legends of each of these quantifications.

For quantifications of percentage of pimonidazole coverage in each organ, regions of tissues were selected. The percentage of pimonidazole coverage was calculated by dividing the pimonidazole-stained area in the numerator by the total area of the selected tissue in the region defined as referent. For quantifications of percentage of leaked dextran coverage in each organ, regions of tissues were selected. The dextran-labeled area outside the vessels was identified and measured. The percentage of leaked dextran was calculated by dividing the dextran-labeled area out of the vessels in the numerator by the total area of the selected tissue.

For the heatmap of the expression of various markers, average intensity analysis on expressions of each marker was applied on the basis of the entire region of each organ using Fiji. According to the highest value and lowest value of the expressions, a range of the intensity value was set. Then, four levels of average expressions for each marker were determined from green to red color and plotted as a heatmap.

### Flow cytometry

For flow cytometry of stem cells in different organs, C57BL/6J mice were used for collecting tissues. Organs were crushed in collagenase A (0.7 mg/ml) (Sigma-Aldrich, 10103578001) and incubated at 37°C for 45 min. Followed by the addition of 100 ml of fetal bovine serum, a single-cell suspension was acquired after filtering and centrifuging, and an equal number of cells were obtained for analysis. Cells were then stained with phycoerythrin (PE)–conjugated CD45 (BioLegend, 103105), PE-conjugated Ter119 (TONBO Biosciences, 50-5921-U025), PE-conjugated CD31 (Sino Biological, 10148-MM13-P), allophycocyanin-conjugated CD140a (BioLegend, 135907), and Brilliant Violet 421–conjugated Sca-1 (BioLegend, 108127) on ice for 2 hours. After washing thoroughly, cells were acquired using the LSRFortessa X-20 flow cytometer, and data were analyzed via BD FACSDiva software (version 6.0, BD Biosciences). Stem cells were quantified as CD140a^+^/Sca-1^+^/CD45^−^/Ter119^−^/CD31^−^.

For flow cytometric analysis of proliferating ECs in different organs, organs from 10-, 30-, and 55-week-old C57BL/6J mice were collected and crushed in collagenase A (0.7 mg/ml) (Sigma-Aldrich, 10103578001) and incubated at 37°C for 45 min. After obtaining the single-cell suspension, an equal number of cells were resuspended in 0.5 ml of PBS and added with 4.5 ml of prechilled 70% cold ethanol (−20°C) in a dropwise manner while gently vortexing to minimize cell aggregation. After incubating at −20°C for 2 hours, ethanol was removed, and cells were rinsed by centrifugation. Cells were stained with rat anti-Emcn antibody (Santa Cruz, sc-65495) and rabbit anti-Ki67 antibody (Abcam, ab15580) on ice for 2 hours and then incubated PE-conjugated donkey anti-rat secondary antibody (Jackson ImmunoResearch, 712-116-153) and Alexa Fluor 647–conjugated donkey anti-rabbit secondary antibody (Jackson ImmunoResearch, 711-606-152) on ice for 1.5 hours. After rinsing two times, cells were acquired using the LSRFortessa X-20 flow cytometer, and data were analyzed via BD FACSDiva software (version 6.0, BD Biosciences). Proliferating ECs were quantified as Emcn positive and Ki67 positive.

For flow cytometry of ROS analysis, organs from different age groups of mice (*n* = 5) were collected in PBS, crushed, and enzymatically dissociated with collagenase A (0.7 mg/ml of PBS) at 37°C for 45 min. The cell suspension was passed through a 40-μm cell strainer. Single-cell suspension was used for flow cytometric analysis after incubating with DHE (2 mM) at 37°C for 10 min. The samples were acquired immediately using the LSR II flow cytometer. The FACS files were analyzed using FlowJo software (version 10.6.1). Cells treated with *tert*-butyl hydroperoxide in PBS (400 mM) for 45 min at 37°C before staining with DHE were used as a positive control.

Thymic epithelial cells (TECs) for flow cytometry were isolated via Liberase (Roche, 11 284 932 001) and deoxyribonuclease I (DNase I; Roche, 5401127001) enzymatic digestion of thymic lobes. Counted cells were stained with anti-CD45 microbeads (Miltenyi Biotec, 130-052-301) for 15 min on ice before negative selection using AutoMACS (Miltenyi Biotec) to enrich for TECs. Samples were then stained for cell surface markers for 20 min at 4°C. For intracellular staining, cells were fixed and permeabilized using the Foxp3 transcription factor staining kit (eBioscience, 00-5523-00) according to the manufacturer’s instructions. Combinations of UEA-1 lectin (Vector Laboratories, L-1060) labeled in-house with Cy5 and the following antibodies were used to stain the cells: CD45 (30-F11, BioLegend, 103128), EpCAM (G8.8, BioLegend, 118220), Ly51 (6C3, BioLegend, 108308), CD80 (16-10A1, BioLegend, 104712), and AIRE (5H12, eBioscience, 53-5934). For flow cytometric analysis of thymocytes, the thymus was crushed, and cells were incubated with antibodies against TCR β (H57-597; eBioscience, 12-5961-83), CD4 (GK1.5; BioLegend, 100414), CD8a (53-6.7; BioLegend, 100730), CD25 (PC61.5; BioLegend, 102036), CD44 (IM7; eBioscience, 61-0441-82), and CD117 (2B8; BioLegend, 105812).

For the assessment of cell viability, DAPI or the LIVE/DEAD Fixable Aqua Dead Cell Stain Kit (Thermo Fisher Scientific, L34957) was used. Stained samples were acquired on a FACSAria III flow cytometer, and the data were analyzed using the FlowJo software (version 10.6.1).

### Vascular leakage assessment

To investigate the vascular leakage status upon aging, wild-type (WT) mice at different ages such as 10, 30, and 55 weeks old were administered with tetramethylrhodamine isothiocyanate–dextran (average molecular weight of 65,000 to 85,000; Sigma-Aldrich, T1162) by tail vein injection at a dose of 20 mg/kg of body weight. Fifteen minutes after injection, mice were euthanized by CO_2_ asphyxiation, and the kidney, thymus, muscle, and spleen were collected for further imaging analysis.

### Enzyme-linked immunosorbent assay

For tissue lysate preparation, 300 ml of complete extraction buffer [100 mM tris (Sigma-Aldrich, T1503), 150 mM NaCl (Sigma-Aldrich, S7653), 1 mM EGTA (Sigma-Aldrich, E4378), 1 mM EDTA (Sigma-Aldrich, E6758), 1% Triton X-100 (VWR, 306324N), and 0.1% sodium deoxycholate (Sigma-Aldrich, D6750)] supplemented with protease inhibitor cocktail (Fisher Bioreagents, 12801640) was added to 5 mg of tissue. Then, samples were homogenized with an electric homogenizer and maintained at constant agitation for 2 hours at 4°C. After centrifuging for 20 min at 13,000 rpm at 4°C, the samples were placed on ice. CDKN2A in the tissue lysate was determined by an ELISA kit (Abcam, ab230131) according to the manufacturer’s instructions. IL-6 was determined by an ELISA kit (Abcam, ab222503) according to the manufacturer’s instructions.

### Quantitative PCR

qPCR was performed using TaqMan gene expression assays on the ABI PRISM 7900HT Sequence Detection System. The FAM-conjugated TaqMan probes were used along with the TaqMan Gene Expression Master Mix (Applied Biosystems, 4369510). Gene expression assays were normalized to endogenous VIC-conjugated Actb probes as standard. All RNA samples were immediately processed for complementary DNA preparation using the SuperScript IV First-Strand Synthesis System (Invitrogen, 18091200). To perform qPCR, FAM-conjugated TaqMan probes were used along with TaqMan Gene Expression Master Mix (Applied Biosystems, 4369510).

### RNA isolation

Freshly dissected thymus, spleen, and kidney from young (8- to 10-week-old) and aged (50- to 55-week-old) mice were subjected to digestion with 0.2% collagenase IV, dispase (1.25 U/ml) (Thermo Fisher Scientific, catalog no. 171055-041), and DNase I (7.5 mg/ml) (Sigma-Aldrich, catalog no. D4527-10KU) for 45 min at 37°C to prepare single-cell suspension for the isolation of ECs. ECs from the single-cell suspension was isolated using a magnetic bead (Invitrogen, 11035)–based separation method using the CD144 and Emcn antibodies. The ECs were isolated from different organs from the batch of the same mice; thus, the different organs and their ECs were derived from the same mice and processed simultaneously. RNA from 150,000 cells from each group was isolated using the RNeasy Plus Micro Kit (QIAGEN, 74034) according to the manufacturer’s instructions. The purity of the MACS (magnetic-activated cell sorting)–sorted ECs was assessed by qPCR analysis of EC markers *Emcn* and *Cdh5* versus pericyte markers *Pdgfrb* and *Cspg4*. Only the samples of purified ECs with high expression of endothelial markers and with undetectable levels of pericyte markers were processed for bulk RNA-seq.

### RNA-seq data analysis

The RNA quality was checked using a 2100 BioAnalyzer (Agilent). The QuantSeq 3′ mRNA-seq library prep kits (Lexogen, 015.24) according to the manufacturer’s instructions were used for the preparation of sequencing libraries. The samples were sequenced in three biological replicates and two technical replicates. The RNA-seq data were uploaded to Array Express with the accession number E-MTAB-8941 (www.ebi.ac.uk/arrayexpress/experiments/E-MTAB-8941/) (username: Reviewer_E-MTAB-8941; password: eo8gB86y).

### Quality assessment

Data quality assessment of raw sequence data was analyzed by FastQC (version 0.11.5). Alignment to reference genome is as follows: The raw reads were mapped to genome assembly (GRCm38) using STAR (version 2.7.3a) with setting the parameter (STAR_2.7.3a) (*54*). The quantification of the aligned reads on a per-gene basis was obtained using HTSeq (version 0.11.0) with the following settings: version: HTSeq- 0.11.0; [htseq-count-mode = intersectionnonempty − stranded = F] (*55*). To explore the similarities and dissimilarities between samples, the count data of technical replicates for the sample were collapsed using the collapse Replicates function; later data were normalized using the Variance Stabilizing Transformation function from the DESeq2 package (Bioconductor, version 3.10) (*56*).

### Differentially expressed gene analysis

The differentially regulated genes were identified using different contrast between young and aged mice using the DESeq2 package (Bioconductor, version 3.10) (*56*). The differentially expressed genes are listed with an FDR-adjusted *P* value cutoff of <0.01. The differentially regulated genes of Ensembl ID were annotated to gene symbols and Entrez Gene using biomaRt (Bioconductor, version 3.10) (*57*) and, further, the differentially regulated genes filter with protein biotype. For visualizing heatmaps, the count data were normalized and converted to count per million (CPM) value using the calNorm factor function in edgeR (Bioconductor, version 3.10) (*58*).

### Gene set enrichment analysis

The overrepresented pathway term was performed using generally applicable gene set enrichment (GAGE; Bioconductor, version 3.10) (*59*). The mouse annotation KEGG was used for this functional annotation. The signaling pathway enrichment analysis was performed on the basis of the one-on-one comparison between samples of young mouse and aged mouse, where young mouse subtype was used as a reference sample, whereas aged mouse cells were taken as an expression dataset. The analysis steps were followed as described in the vignettes of GAGE (Bioconductor, version 3.10) (*59*). Here, we explored the expression changes in one direction (either up- or down-regulation) in the gene sets. The significance of enrichment test was calculated by a two-sample *t* test, *P* values were adjusted for multiple testing using the Benjamini and Hochberg method, and a *q* value of <0.1 was considered as significant.

### Image database

3D image database as shared resources are freely available. Images can be easily accessed via an OMERO web interface (*60*), which has basic analysis capabilities and the ability to download image sets for further analyses such as 3D surface rendering and interactions between different cell types. OMERO.iviewer can be used to open and browse these multichannel images. Briefly, double-click the “full viewer” button to open an image in a larger viewer. Images can be zoomed in and out by scrolling the mouse wheel and can be rotated by holding “shift” on the keyboard and then dragging the mouse. To change the Lookup Table (LUT) of a particular channel, click on the downward-facing arrow next to the channel label. The intensity of the channel can be also inverted by checking the checkbox on the top. Maximum intensity projection of a z-stack can be made by clicking the “stack” icon in the bottom left corner of the central pane. The user interface documentation is at https://omero-guides.readthedocs.io/en/latest/iviewer/docs/iviewer_viewing.html. The link to access image database is http://homeros.kennedy.ox.ac.uk/pub/chen-et-al-2021-3dOrgans.

### Statistical analysis

All statistical analyses were conducted with GraphPad Prism software (version 8.02) and OriginPro (version 9.1). All data are expressed as means ± SD. Two-tailed Student’s unpaired *t* test was used to determine the significance of the difference between means of two groups. One-way analysis of variance (ANOVA) test with Tukey’s multiple comparisons test was applied for the analysis of the statistical significance of differences between more than two groups. *P* < 0.05 was considered significant. ns indicates not significant; *P < 0.05; **P < 0.01; ****P < 0.001; ****P < 0.0001. For combined box and whiskers and scatterplot, the box represents means ± SD, the line in the box represents the median, the lower and upper lines display the minimum and the maximum of the values, and the line on the right side of this combo represents the sample distribution. All the groups of each experiment contained balanced number of male and female subjects. Multiple and independent experiments were performed to validate the reproducibility of findings.

## SUPPLEMENTARY MATERIALS

Supplementary material for this article is available at http://advances.sciencemag.org/cgi/content/full/7/6/eabd7819/DC1

## Appendix

View/request a protocol for this paper from *Bio-protocol*.
